# Supplementary material for: A hotspot of Toxoplasma gondii Africa 1 lineage in Benin: How new genotypes from West Africa contribute to understand the parasite genetic diversity worldwide
Source: PLoS Negl Trop Dis. 2021 Feb 11;15(2):e0008980. doi: 10.1371/journal.pntd.0008980 (PMC7904144; doi:10.1371/journal.pntd.0008980)
Supplement: Attachment — Submitted filename: Response to reviewers.pdf [file pntd.0008980.s002.pdf]

Azra Hamidović
UMR Inserm 1094
Neuroépidémiologie Tropicale
2 rue du Dr Marcland
87025 Limoges Cedex, France
Phone : +33629913722
azra.hamidovic@unilim.fr

Limoges, September 25th 2020

Editor, *PLOS Neglected Tropical Diseases*

Dear editor, dear reviewers,

We thank you for reviewing our manuscript now entitled "**A hotspot of *Toxoplasma gondii* Africa 1 lineage in Benin: How new genotypes from West Africa contribute to understand the parasite genetic diversity worldwide**" (PNTD-D-20-01195) submitted to PLOS Neglected Tropical Diseases.

We have taken into account the various comments and produced a revised version of the manuscript. We highlighted all the modifications in the manuscript (Manuscript_Rev.doc) and in S1 Table (S1 Table_Rev). As asked, we uploaded the same files without highlights as final versions.

Also, please find below answers to specific reviewers questions.

We hope that you will find this new version suitable for publication in PLOS Neglected Tropical Diseases.

Respectfully,

On behalf of all co-authors, Azra Hamidović

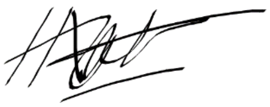

Reviewer's Responses to Questions

Key Review Criteria Required for Acceptance?

As you describe the new analyses required for acceptance, please consider the following:

Methods

-Are the objectives of the study clearly articulated with a clear testable hypothesis stated?

-Is the study design appropriate to address the stated objectives?

-Is the population clearly described and appropriate for the hypothesis being tested?

-Is the sample size sufficient to ensure adequate power to address the hypothesis being tested?

-Were correct statistical analysis used to support conclusions?

-Are there concerns about ethical or regulatory requirements being met?

Reviewer #1: yes

Reviewer #2: The Methods in this manuscript look good, and suitable for the proposed study. However, I do have some comments on some sections:
i) in lines 150 to 151, the authors mention that they collected the hearts and brains of seropositive animals. Why they collected the hearts and not some other muscular tissue that Toxoplasma is known to be found in infected animals?
Hearts and brains are known to be organs of tropism for *T. gondii* isolation in chickens (Dubey et *al.* Veterinary Parasitology 214 (2015) 55–58). Although it is possible to isolate the parasite from muscles, the parasite concentration is higher in the heart and brain and the organs smaller, enhancing the chance of a successful isolation.

ii) in section "Mouse virulence" (lines 198 to 206) there is no mention in the analysis of cysts in infected animals though this data is mentioned in the Results section. How the number of cysts was evaluated? How many mice were used per condition? Which mouse strain was used?
Cysts are not mentioned here because the mouse virulence was evaluated only with Africa 1 strains which usually only provide ascites with tachyzoites (the virulence is so high that parasites does not have the time to encyst in the brain).
As for the protocol, we followed the description by Galal and collaborators, as indicated lines 152-153: three Swiss mice per sample and a daily monitoring. At the end of the four weeks of the isolation protocol, we do a serology on surviving mice (which in our case only happened for Type III and Africa 4 strains). Seropositive mice were euthanized and brain samples were homogenized with 1 ml of physiological solution for microscopic examination of tissue cysts. We count the number of cysts to confirm a successful isolation, and it helps us know how many aliquots of one sample will be done for the cryogenisation process. We do not link it to virulence, since no virulence was observed in this case.

Results

-Does the analysis presented match the analysis plan?

-Are the results clearly and completely presented?

-Are the figures (Tables, Images) of sufficient quality for clarity?

Reviewer #1: yes

Reviewer #2: Results were clearly presented by the authors.
In the section "Isolation, virulence and genotyping of T gondii isolates from Benin" (lines 214 to 221), the authors mentioned that they evaluated the number of cysts in the brain of laboratory mice. As I mentioned in the "Results" section, there is no mention on how this was evaluated. Here, in the "Results" the authors should comment how many cysts were produced with the strains they isolated. Maybe a table comparing the number of isolated cysts per brain per mice from the different strains.
Only two isolated strains (the Type III clone) were non-virulent (two seropositive mice out of three for each strain) and among those four mice, only three had cysts in their brain, respectively 10, 16 and 3 cysts per 25µL of homogenate. Since we have a volume of 200µL for each brain, we can deduce that the total of cysts is 80, 128 and 24 per brain. We added this information at lines 219-220.

Conclusions

-Are the conclusions supported by the data presented?

-Are the limitations of analysis clearly described?

-Do the authors discuss how these data can be helpful to advance our understanding of the topic under study?

-Is public health relevance addressed?

Reviewer #1: yes

Reviewer #2: The authors make a good discussion of the results. On minor note, the authors suggest that the lack of house mice might be connected to the absence of Africa 1 strain, making the Type II strain more common. This would suggest that Type II strain was not found before in Africa, being brought by European colonisers. Maybe the authors could explore more this part, as well as the connection of African strains with Brazilians ones.
Actually, the lack of house mouse is linked to the absence of Type II (and vice versa). The type II strains were detected in Senegal (lines 354, and 382-4, from Galal et *al.* 2019), but since it is not the case in Benin, we tried to not speculate too much on this part.
As for the connection between African and Brazilians strains, we discuss this link in the discussion (starting line 416 to line 437).

The authors mentioned in the Introduction (lines 87-88) that ocular toxoplasmosis was important in West and Central Africa. Would these strains be connected to the Brazilians ones that cause/are important in ocular toxoplasmosis in Brazil?
Brazilian (or South American) strains responsible for ocular toxoplasmosis are usually “atypical” strains, with some alleles common with Type I and Type III strains. Atypical strains and Africa 1 strains are very different and are classified in different phylogenetic groups. Even though they both share common virulence allele with Type I, we did not want to speculate too much on this part. An interesting perspective would be to use genomics to identify introgressions in *T. gondii* genomes of strains from these two continents.

One final comment is about a phrase in "Introduction" (lines 106-111), where the authors give the impression that there was no Toxoplasma in Africa before European colonisers started to port in the shores of African countries. Is there evidence of this? If so, they should include the reference.
Toxoplasma was present in Africa way before colonization. A recent pre-print revealed that *Rickettsia felis* and *Toxoplasma gondii* were implicated in the death of a hunter-gatherer boy from South Africa

2,000 years ago (Rifkin et *al.*, https://www.researchgate.net/publication/343188075) (we added this citation line 103).
The formulation of this sentence is probably confusing. Here, we hypothesize that new lineages could have emigrated through exchanges. We added "non-autochtonous lineages of *T. gondii*" to the sentence (lines 105-106).

Editorial and Data Presentation Modifications?

Use this section for editorial suggestions as well as relatively minor modifications of existing data that would enhance clarity. If the only modifications needed are minor and/or editorial, you may wish to recommend "Minor Revision" or "Accept".

Reviewer #1: minor revision

Reviewer #2: (No Response)

Summary and General Comments

Use this section to provide overall comments, discuss strengths/weaknesses of the study, novelty, significance, general execution and scholarship. You may also include additional comments for the author, including concerns about dual publication, research ethics, or publication ethics. If requesting major revision, please articulate the new experiments that are needed.

Reviewer #1: This is an extension of the concept published earlier in PLOS-Neglected Tropical Diseases from another African country. This is a thorough investigation and I have only minor comments.

1. Line 95 replace nowaday with currently
It has been changed.
2. Line 138 replace animals with poultry
It has been replaced.
3. Line 142- replace insulin needle with—provide dimension
We replaced it by "syringe with a 25G needle".
4. Line 150-delete by bleeding
It has been deleted.
5. PROVIDE a SUPPLENTARY TABLE-giving full details of poultry surveyed-species, source, domestic/wild, antibody titer, adult/juvenile, bioassay details, strain preserved, isolate designation
Our sampling is only on domestic animals, with a predominance of chickens (98% of the sampling). As for epidemiology criteria such as antibody titer, age, presence of cats in the environment and type of soil, a second paper with a more extensive animal blood sampling including this one (without isolation) is in preparation with Beninese collaborators. However, we decided to add the species and localization to S1 Table.
6. Line 203delete thanks to a qpcr—
It has been changed.
7. Line 210—list seroprevalence for each species
Since this article did not focus on seroprevalence, we did not include that information. A second paper with a more extensive animal blood sampling including this one (without isolation) is in preparation with Beninese collaborators.
8. Line 233-no cyst by direct microscopic examination of brain for tissue cysts
We replaced it by "no brain cyst by direct microscopic examination".
9. Line 313 delete—hardly access----with a remote area
It has been changed.
10. Line 316-space before the
We added it.
11. Line 323—no personal communication-Galal is coauthor

It has been changed.
12. Line330—delete but-------optimixe
We think it is important that we keep this part to support our argument.
13. Replace genic with genetic throughout
We kept “genic” since we used the genic differentiation with Genepop, which enables us to study the distribution of alleles in the various samples.
14. Line 385 need reference for Galal
The reference was added.
15. Line 446delete-thanks----
It has been deleted.
16. Line303-specify extra dozens
We changed it for “39 T. gondii genotypes”.

We thank you for your time, comments and suggestions to improve our manuscript.

Reviewer #2: The manuscript is well written and the authors clearly present their ideas and results. The Discussion is well balanced and the results properly discussed with published literature. Overall, it is a well-sounded manuscript.literature. Overall, it is a well-sounded manuscript.
We thank you for your points of discussion, comments and appreciation of our manuscript.
